# After the Fort McMurray wildfire there are significant increases in mental health symptoms in grade 7–12 students compared to controls

**DOI:** 10.1186/s12888-018-2007-1

**Published:** 2019-01-10

**Authors:** Matthew R. G. Brown, Vincent Agyapong, Andrew J. Greenshaw, Ivor Cribben, Pamela Brett-MacLean, Julie Drolet, Caroline McDonald-Harker, Joy Omeje, Monica Mankowsi, Shannon Noble, Deborah Kitching, Peter H. Silverstone

**Affiliations:** 1 Department of Computing Science, University of Alberta, Edmonton, Canada; 2 Department of Psychiatry, University of Alberta, Edmonton, AB T6G 2B7 Canada; 3 Alberta School of Business, University of Alberta, Edmonton, Canada; 4 Faculty of Social Work, University of Calgary, Calgary, Canada; 5 Department of Sociology and Anthropology, Mount Royal University, Calgary, Canada; 6 Fort McMurray Catholic School District, Fort McMurray, Canada; 7 Fort McMurray Public School District, Fort McMurray, Canada

**Keywords:** Youth, Mental health, Wildfire, Disaster, Depression, Anxiety, PTSD, Substance use, Self-esteem, Resilience

## Abstract

**Background:**

In order to examine the impact of disasters on adolescent mental health, this study compared population mental health survey data from two communities in Alberta, Canada: Fort McMurray, which experienced a major natural disaster, and Red Deer, which did not.

**Methods:**

Data from 3070 grade 7–12 students from Fort McMurray, Alberta, Canada (collected in 2017, 18 months after the 2016 wildfire) was compared with data from 2796 grade 7–12 students from Red Deer, Alberta, Canada (collected in 2014). The same measurement scales were used for both surveys. Both of these cities have populations of approximately 100,000, and both cities are located in Alberta, Canada. For this reason, Red Deer is an appropriate non-disaster impacted community to compare to the disaster impacted community of Fort McMurray.

**Results:**

The results of this comparison demonstrate that mental health symptoms were statistically significantly elevated in the Fort McMurray population when compared to the control population in Red Deer. This occurred for scores consistent with a diagnosis of depression (31% vs. 17%), moderately severe depression (17% vs. 9%), suicidal thinking (16% vs. 4%), and tobacco use (13% vs. 10%). Consistent with there being major mental health impacts from the 2016 Fort McMurray wildfire, self-esteem scores and quality of life scores were also statistically significantly lower in Fort McMurray. While the rates of anxiety disorder were similar (15% vs. 16%), the mean scores on the anxiety scale were slightly higher, with this difference reaching statistical significance. There were no statistical differences in the rates or scores for alcohol or substance use.

**Conclusions:**

Our results are consistent with previous findings showing a significant negative impact of disasters on many aspects of adolescent mental, with a particular increase in symptoms related to depression and suicidal thinking. These findings highlight first, the need to identify adolescents most at risk of developing psychiatric symptoms after experiencing the trauma of disaster and second, the importance and necessity of implementing short and long term mental health intervention programs specifically aimed at adolescents, in order to help mitigate the negative effects of disasters on their mental health.

**Electronic supplementary material:**

The online version of this article (10.1186/s12888-018-2007-1) contains supplementary material, which is available to authorized users.

## Background

On May 3, 2016, the population of 88,000 living in Fort McMurray, Alberta, Canada was evacuated due to a major wildfire. The fire, dubbed “The Beast” in the popular media [1], destroyed 10% of the homes in Fort McMurray and spread across 590,000 ha of land before being contained. A phased re-entry of residents began in early June 2016. According to the Insurance Bureau of Canada, the cost of the Fort McMurray wildfire was estimated at CAD$3.6 billion, the most expensive insured catastrophe in Canadian history [2]. Extensive damage was caused to local infrastructures including homes, schools, and local businesses. Today, just two years post-wildfire, many individuals continue to be impacted by not only the physical damage caused to the community but also the social, emotional, and psychological difficulties that often occur in the aftermath of disaster [3]. Adolescents are particularly affected by disasters because of their dependence on adults and physical, psychological, and social factors related to their developmental stage. However, only a small number of studies have investigated the effects of natural disaster on adolescent mental health and well-being, either by comparing population mental health in disaster impacted versus non-disaster impacted communities or by comparing members of a disaster impacted community with differing levels of traumatic exposure. Previous studies suggest that wildfires have a negative impact on the mental health of residents of the affected areas (see [4] for review). Wildfires are unique in that their effects persist for an extended duration of time, thereby furthering the disruption of day-to-day functioning and ultimately often leading to reduced psychological adjustments and overall well-being [5, 6]. After wildfires, children and adolescents exhibit increased incidence of post-traumatic stress disorder (PTSD) and depression [7, 8]. Studies conducted with both adults and children/adolescents report that both have an increased incidence of PTSD [9] as well as increased symptoms of depression and stress [10]. Studies focusing only on adults show that they exhibit increased symptoms of depression and anxiety [11, 12], increased incidence of PTSD [11, 13], increased levels of psychological distress [14], and increased consumption of anxiolytics-hypnotics [15].

More broadly, non-wildfire disasters, such as floods, earthquakes, and tsunamis, also have a negative impact on mental health, as reviewed in Goldmann and Galea [16], Kar [17], and Norris et al. [18, 19]. Briefly, disasters are associated with increased incidence of major depressive disorder, generalized anxiety disorder, PTSD, and substance use disorder in children and adults. A link between disasters and increased depression is also supported by Tang et al. [20]. One more recent study associates earthquakes with increased incidence of PTSD [21]. Moreover, child and adolescent pre-existing mental health challenges are often exacerbated following exposure to disaster [22]. This is particularly concerning given that individuals’, particularly adolescents’, coping efficacy is an important mediating factor in the post-disaster recovery process and can have a significant impact on long-term mental health outcomes (also see Weems and Graham [23] on differential PTSD symptom recovery trajectories in adolescents exposed to hurricanes).

Using data from a battery of self-report questionnaires, the current study investigated population mental health effects in the form of depression, suicidal thinking, anxiety, alcohol/substance use disorder, tobacco use, self-esteem, and quality of life in the population of grade 7–12 students in Fort McMurray, Alberta following the wildfire. This data was compared to a sample of grade 7–12 students from Red Deer, Alberta who participated in a previously-conducted study “Empowering a Multimodal Pathway Towards Healthy Youth” (EMPATHY) in 2014. The EMPATHY study included the same mental health questionnaires [24, 25]. The Red Deer, Alberta student population served as the non-disaster impacted control group. Red Deer provides a good comparison to Fort McMurray as both cities are located in Alberta, Canada (Central and Northern Alberta, respectively); both have similar socioeconomic distributions; and both have populations of approximately 100,000 (Fort McMurray had a population of 88,000 in May 2016; Red Deer had a population of 98,600 in 2014 at the time of the EMPATHY study).

The current work investigates the effects of wildfire on mental health in adolescents with a sample size (*n* = 3070 from Fort McMurray; *n* = 2976 from Red Deer) that is larger than in any previous study, to our knowledge. This paper adds to a small but important literature on the impact of wildfires and other disasters on adolescent mental health, with implications for policy and service delivery in the aftermath of the 2016 Fort McMurray wildfire and more generally.

Based on previous research, our hypotheses were that students in the post-wildfire community of Fort McMurray would exhibit elevated symptoms of depression, suicidal thinking, anxiety, alcohol/substance misuse, and tobacco use, in addition to lower scores for self-esteem and quality of life in comparison to Red Deer.

## Methods

### Overview and ethical considerations

The data from Fort McMurray was collected in November 2017, 18 months after the 2016 wildfire. The two school boards in Fort McMurray – Fort McMurray Public Schools and Fort McMurray Catholic Schools – surveyed 3252 students in grades 7–12 in the community in order to evaluate the effectiveness of the school mental health and support programs put in place to assist students after the wildfire. The survey consisted of a battery of ten questionnaires assessing aspects of demographics, mental health, resilience, and the impact of the 2016 wildfire (details below). Researchers from the University of Alberta were asked to collaborate and provide assistance in designing the survey questionnaires and analyzing the survey data.

All survey data was collected under the auspices and ethical guidelines of the two Fort McMurray school systems. The survey was administered as part of their standard curriculum and as an ongoing assessment of the educational and support programs they had put in place after the wildfire (see Additional file 1: Appendix for list of programs). The selection of measurement instruments was determined by the school systems and was informed by the existing scholarly literature and findings. Parents and guardians were notified of the process by written letter two weeks prior to the administration of the survey in the schools, and they had the option to opt their child(ren) out of the survey. Students had the option to participate or not in the survey, and this was explained at the start of each survey data collection session (see details below). Survey participation was anonymous; participants were not asked for their names. After the data was collected, the anonymized data was made available for analysis by researchers from the University of Alberta. The analysis of the survey data was approved by the University of Alberta’s Health Research Ethics Board on June 26th, 2017 (ethics protocol number Pro00072669).

The data from Red Deer was collected through the previous EMPATHY project, which surveyed population mental health indicators in public schools in Red Deer using seven questionnaires. Six of the seven questionnaires used in Red Deer were also used in Fort McMurray. The seventh questionnaire used in Red Deer was a demographics questionnaire. The Fort McMurray survey also included a demographics questionnaire, which was somewhat different from the Red Deer one. Full details of the EMPATHY project are available [24, 25]. In this paper, we focus on the six questionnaires that were used in both the communities of Fort McMurray and Red Deer, using Red Deer as a control population. Ethics considerations for data collection in the Red Deer EMPATHY project have been described in detail previously [24, 25] but were similar to those for the Fort McMurray data collection. The EMPATHY program itself was approved by the Health Research Ethics Committee of the University of Alberta on December 5th, 2013, ethics protocol number Pro00041063.

This paper provides a comparison and analysis of the survey data collected from grade 7–12 students in the disaster impacted community of Fort McMurray and the non-disaster impacted community of Red Deer, in order to examine the effects of disasters on the mental health of adolescents.

### Survey questionnaires

Questionnaire details are shown in Table 1. Both the Fort McMurray and Red Deer surveys included a questionnaire on demographics, though the precise questions differed between the two sites (see Table 1 for details).

**Table 1:** – Questionnaire details

	Questions	Answer choices
Ft McMurray Demographics Questionnaire		
1	Are you at school right now, while you are taking the survey?	Yes, no
2	Are you a student?	Yes, no
3	What gender do you identify with?	Female, male, other, prefer not to say
4	What is your age in years?	10 years or less, 11 years, 12 years, 13 years, 14 years, 15 years, 16 years, 17 years, 18 years, 19 years, 20 years or more
5	What is your school?	7, 8, 9, 10, 11, 12, other
6	What grade are you in?	Select from a list of all Ft McMurray schools with any classes in grades 7–12
7	What school were you in for grade 6?	Select from a list of all Ft McMurray schools with grade 6
Red Deer EMPATHY Demographics Questionnaire		
1	What gender do you identify with?	Female, male
2	What is today’s date?	Date selection
3	What is your date of birth?	Date selection
4	What grade are you in?	6, 7, 8, 9, 10, 11, 12
Ft McMurray Patient Health Questionnaire (PHQ-A, Depression Symptoms)		
	Over the past 2 weeks, how often have you been bothered by any of the following problems?	
1	Feeling down, depressed, irritable or hopeless	Not at all, Several days, More than half the days, Nearly every day
2	Little interest or pleasure in doing things?	Same as above
3	Trouble falling or staying asleep, or sleeping too much	Same as above
4	Poor appetite, weight loss, or overeating?	Same as above
5	Feeling tired, or having little energy?	Same as above
6	Feeling bad about yourself-or that you are a failure or that you have let yourself or your family down	Same as above
7	Trouble concentrating on things, such as school work, reading or watching television	Same as above
8	Moving or speaking so slowly that other people could have noticed. Or the opposite-being so figety or restless that you have been moving around a lot more than usual	Same as above
9	Thoughts that you would be better off dead, or of hurting yourself in some way	Same as above
	Questions 10 and 11 asked only if answer to question 9 is not “Not at all”	
10	Has there been a time in the past month when you have had serious thoughts about ending your life?	Yes, no
11	Have you ever, in your WHOLE LIFE, tried to kill yourself or made a suicide attempt?	Yes, no
Red Deer EMPATHY Patient Health Questionnaire (PHQ-A, Depression Symptoms)		
	Over the past 2 weeks, how often have you been bothered by any of the following problems?	
1	Feeling down, depressed, irritable or hopeless	Not at all, Several days, More than half the days, Nearly every day
2	Little interest or pleasure in doing things?	Not at all, Several days, More than half the days, Nearly every day
3	Trouble falling or staying asleep, or sleeping too much	Not at all, Several days, More than half the days, Nearly every day
4	Poor appetite, weight loss, or overeating?	Not at all, Several days, More than half the days, Nearly every day
5	Feeling tired, or having little energy?	Not at all, Several days, More than half the days, Nearly every day
6	Feeling bad about yourself-or that you are a failure or that you have let yourself or your family down	Not at all, Several days, More than half the days, Nearly every day
7	Trouble concentrating on things, such as school work, reading or watching television	Not at all, Several days, More than half the days, Nearly every day
8	Moving or speaking so slowly that other people could have noticed. Or the opposite-being so figety or restless that you have been moving around a lot more than usual	Not at all, Several days, More than half the days, Nearly every day
9	Thought of hurting yourself in some way	Not at all, Several days, More than half the days, Nearly every day
10	Thoughts that you would be better off dead	Not at all, Several days, More than half the days, Nearly every day
11	If you checked off “any problems”, how difficult have these problems made it for you to do your work, take care of things at home, or get along with other people?	Not difficult at all; Somewhat difficult; Very difficult; Extremely difficult
	Questions 12 and 13 asked only if answer to question 10 is not “Not at all”	Yes, no
12	Has there been a time in the past month when you have had serious thoughts about ending your life?	Yes, no
13	Have you ever, in your WHOLE LIFE, tried to kill yourself or made a suicide attempt?	Yes, no
Hospital Anxiety and Depression Scale (HADS, Anxiety Symptoms)		
	Tick the box beside the reply that is closest to how you have been feeling in the past week. Don’t take too long over you replies: your immediate is best.	
1	I feel tense or wound up:	Most of the time; A lot of the time; From time to time, occasionally; Not at all
2	I get a sort of frightened feeling as if something bad is about to happen:	Very definitely and quite badly; Yes, but not too badly; A little, but it doesn’t worry me; Not at all
3	Worrying thoughts go through my mind:	A great deal of the time; A lot of the time; From time to time, but not too often; Only occasionally
4	I can sit at ease and feel relaxed:	Definitely; Usually; Not often; Not at all
5	I get a sort of frightened feeling like ‘butterflies’ in the stomach:	Not at all; Occasionally; Quite often; Very often
6	I feel restless and have to be on the move:	Very much indeed; Quite a lot; Not very much; Not at all
7	I get sudden feelings of panic:	Very often indeed; Quite often; Not very often; Not at all
CRAFFT Questionnaire (Drugs/Alcohol/Tabacco)		
	During the past 12 months, did you:	
1	Drink any alcohol (more than a few sips)?	Yes, no
2	Smoke any marijuana or hashish?	Yes, no
3	Use anything else to get high?	Yes, no
4	Have you ever ridden in a CAR driven by someone (including yourself) who was “high” or had been using alcohol or drugs?	Yes, no
	Questions 5–9 asked only if “yes” to one or more of questions 1–3.	
5	Do you ever use alcohol or drugs to RELAX, feel better about yourself, or fit in?	Yes, no
6	Do you ever use alcohol or drugs while you are by yourself, or ALONE?	Yes, no
7	Do you every FORGET things you did while using alcohol or drugs?	Yes, no
8	Do your FAMILY or FRIENDS ever tell you that you should cut down on your drinking or drug use?	Yes, no
9	Have you ever gotten into TROUBLE while you were using alcohol or drugs?	Yes, no
Tobacco Use Questionnaire		
	During the past month:	
1	Do you smoke tobacco products?	Yes, no
2	Do you use smokeless tobacco products?	Yes, no
Rosenberg Self-Esteem Scale		
1	On the whole, I am satisfied with myself.	Strongly agree, Agree, Disagree, Strongly disagree
2	At times, I think I am no good at all.	Strongly agree, Agree, Disagree, Strongly disagree
3	I feel that I have a number of good qualities.	Strongly agree, Agree, Disagree, Strongly disagree
4	I am able to do things as well as most other people	Strongly agree, Agree, Disagree, Strongly disagree
5	I feel I do not have much to be proud of.	Strongly agree, Agree, Disagree, Strongly disagree
6	I certainly feel useless at times.	Strongly agree, Agree, Disagree, Strongly disagree
7	I feel that I’m a person of worth, at least on an equal plane with others.	Strongly agree, Agree, Disagree, Strongly disagree
8	I wish I could have more respect for myself.	Strongly agree, Agree, Disagree, Strongly disagree
9	All in all, I am inclined to feel that I am a failure.	Strongly agree, Agree, Disagree, Strongly disagree
10	I take a positive attitude toward myself.	Strongly agree, Agree, Disagree, Strongly disagree
Kidscreen Questionnaire (Quality of Life)		
	Thinking about the last week:	
1	Have you physically felt fit and well	Not at all, slightly, moderately, very,
2	Have you felt full of energy?	Never, seldom, quite often, very often,
3	Have you felt sad?	Never, seldom, quite often, very often,
4	Have you felt lonely?	Never, seldom, quite often, very often,
5	Have you had enough time for yourself?	Never, seldom, quite often, very often,
6	Have you been able to do the things that you want to do in your free time?	Never, seldom, quite often, very often, always
7	Have your parent(s) treated you fairly?	Never, seldom, quite often, very often,
8	Have you had fun with your friends?	Never, seldom, quite often, very often,
9	Have you got on well at school?	Not at all, slightly, moderately, very,
10	Have you been able to pay attention?	Never, seldom, quite often, very often,
11	In general, how would you say your health is?	Excellent, very good, good, fair, poor

The surveys in both Fort McMurray and Red Deer shared the following six questionnaires:

1. The Patient Health Questionnaire, Adolescent version (PHQ-A) assesses symptoms of depression and suicidality [26, 27]. This questionnaire provides a score for depression symptom severity from 0 to 36. In this scale, there were 11 questions in the Fort McMurray version compared to 13 questions in the Red Deer EMPATHY version. This is because the Fort McMurray and Red Deer surveys used slightly different versions of the PHQ-A (see details in Table 1). In particular, question #9 in the Fort McMurray version was split into two questions (#9 and 10) in the Red Deer version. To enable use of the same analytical procedure for both sites, we averaged the answers to the Red Deer PHQ-A questions #9 and 10 and rounded to the nearest integer to derive an imputed answer to the Fort McMurray version’s question #9. Question #11 in the Red Deer version of the PHQ-A was not present in the Fort McMurray version, and question #11 in the Red Deer version was, therefore, not included in any of this paper’s analyses.
2. The Hospital Anxiety and Depression Scale (HADS, 7 questions, anxiety-related questions only) assesses symptoms of anxiety [28]. This questionnaire provides a score for anxiety symptom severity from 0 to 21.
3. The CRAFFT Questionnaire (CRAFFT, 9 questions) assesses symptoms of alcohol and substance misuse [29, 30]. This questionnaire provides a score of alcohol / substance misuse severity from 0 to 6.
4. Tobacco Use Questionnaire (2 questions) includes two questions on tobacco use: “Over the past month: Do you smoke tobacco products? Do you use smokeless tobacco products?” Note that the Red Deer version asks the two questions “Over the past 12 months” as opposed to past month.
5. The Rosenberg Self-Esteem Scale (Rosenberg, 10 questions) assesses self-esteem [31]. This questionnaire provides a self-esteem score from 0 to 30.
6. The Kidscreen Questionnaire (Kidscreen-10, 11 questions) assesses quality of life [32]. This questionnaire provides a quality of life score from 0 to 44.

The order of presentation of the six above questionnaires was randomized for each participant, in both the Red Deer and Fort McMurray surveys, to avoid possible sequence effects whereby earlier questionnaires might have influenced participants’ answers to later questionnaires.

In addition, the Fort McMurray survey included the following three questionnaires, which are not analyzed here as they were not administered in the Red Deer EMPATHY project survey: The Impact of Fire Questionnaire (6 questions), a custom questionnaire assessing the impact of the 2016 wildfire on the participant; the Child PTSD Symptom Scale (CPSS, 19 questions [33]; and the Child and Youth Resilience Measure (CYRM-12, 12 questions) [34]. For the Fort McMurray survey, the three additional questionnaires were asked after the six questionnaires that were shared with the Red Deer survey, to prevent the additional questionnaires from affecting answers in any of the six shared questionnaires.

Thus, the shared questionnaires were identical between the two study sites except for minor differences in the demographics, PHQ-A, and tobacco use questionnaires, as noted above.

### Survey administration procedure

In Fort McMurray, students participated in the survey using a laptop or desktop computer during regular school hours in almost all cases. Depending on the school, students either came to a computer laboratory or used laptops brought to their classroom. At the beginning of each survey session, the students were read a script (reproduced in the Additional file 1: Appendix) explaining the purpose of the survey, how to complete the survey, and guaranteeing participant confidentiality, anonymity, and voluntary participation. Students were also given an opportunity to ask questions before participating. Survey participation was anonymous, and the survey did not ask participants for their names. (A small number of students with special circumstances participated from home, using their own computers. These students were given a written version of the same script.) A total of 96 questions were asked in the survey battery used in Fort McMurray. Participation took most students less than 20 min, with a small number of students taking as long as 50 min. Participants were able skip questions, although in the script they were encouraged to answer all questions.

In Red Deer, students participated in the survey using a mobile app on tablets provided by the research team. Full details have been reported previously [24, 25].

### Cut-off scores and probable diagnoses

It is recognized that any scores on a specific scale are not diagnostic. Previously-published cut-off points for probable diagnoses were utilized for the specific scales. These included measures of depression (from PHQ-A), anxiety (from HADS), and alcohol/substance use disorder (from CRAFFT). We use the term “probable diagnosis” because scores were based on self-report scales, not psychiatric clinical interviews. Nonetheless, existing literature reports substantial correspondence between psychiatric clinical diagnoses of depression, anxiety, PTSD, and alcohol/substance use disorder with probable diagnoses based on widely published cut-off scores for the above three sales [29, 30, 35–37]. Each of the three probable diagnoses was defined based on a threshold value for the appropriate scale. Thus, probable depression was defined as having a PHQ-A score of 11 or more [36]. Probable moderately severe depression was defined as having a PHQ-A score of 15 or more [35]. Probable anxiety was defined as having a HADS score of 11 or more [37]. Probable alcohol/substance use disorder was defined as having a CRAFFT score of 2 or more [29, 30]. Tobacco use was defined as answering “yes” to one or both of the two questions in the Tobacco Use Questionnaire. We also defined an “Any of 3 probable diagnoses” criterion as being positive for one or more of three probable diagnoses: depression, anxiety, alcohol/substance use disorder.

Suicidal thinking was defined, for Fort McMurray participants, as selecting the answer “Several days”, “More than half the days”, or “Nearly every day” to the 9th PHQ-A question: “Over the past 2 weeks, how often have you been bothered by any of the following problems? Thoughts that you would be better off dead, or of hurting yourself in some way?” as well as answering “Yes” to the 10th PHQ-A question: “Has there been a time in the past month when you have had serious thoughts about ending your life?” For Red Deer participants, suicidal thinking was defined as an imputed question #9 answer of 1–3 (i.e. equivalent to “Several days”, “More than half the days”, or “Nearly every day”) as well as answering “Yes” to the question “Has there been a time in the past month when you have had serious thoughts about ending your life?”. See Survey Questionnaires section above for differences in PHQ-A between Fort McMurray and Red Deer as well as imputation of answer to question #9 for Red Deer PHQ-A data.

PTSD symptom data from the CPSS was available only for the Fort McMurray students. We took a CPSS score of 15 or more as indicating probable diagnosis of PTSD [38].

### Statistical analysis

We compared participants from Fort McMurray vs. Red Deer on each of the following 12 measures including 1) mean PHQ-A score, 2) mean HADS score, 3) mean CRAFFT score, 4) mean Rosenberg score, 5) mean Kidscreen score, 6) percent probable depression, 7) percent probable moderately severe depression, 8) percent suicidality thinking, 9) percent probable anxiety, 10) percent probable alcohol/substance use disorder, 11) percent tobacco use, and 12) percent any of three probable diagnoses (probable depression, anxiety disorder, or alcohol/substance use disorder). Details of questionnaires and probable diagnoses are given above. For a given measure (eg: mean PHQ-A), only those participants who fully answered each specific relevant questionnaire or scale without skipping any questions were included in the analysis.

We used permutation testing for all statistical comparisons (#iterations = 10^5^). Permutation testing is non-parametric and was chosen for its robustness against non-normality. All tests were two-sided two-sample tests, with a null hypothesis of no difference between the means of the two groups for the given test. In total, we performed 12 individual statistical tests. Multiple comparisons were addressed using the Benjamini-Hochberg method for false discovery rate (FDR) correction. FDR correction indicated a correction threshold of *p* = 0.021. All analyses were done using in-house code built in the Clojure programming language (http://clojure.org).

Effect sizes reported in tables are Cohen’s d (mean difference divided by pooled standard deviation).

There was a small but statistically significant difference in mean age between the Fort McMurray and Red Deer participants (see Demographics section in Results for full details). To exclude possible effects of age on the comparisons of interest, we re-ran all of the comparisons on a subset of the data, subsampled so as to make the distributions of ages as similar as possible between the subsampled Fort McMurray and Red Deer groups (including no significant difference in mean age). This subsampling did not qualitatively change the significant results. In addition, we re-ran all of the comparisons using the full sample of participants, after subtracting out participant age from the measure of interest. This procedure also did not qualitatively affect the significant results.

## Results

The survey in Fort McMurray was administered to the entire population of grade 7–12 students in Fort McMurray. Five Public schools and two Catholics schools were involved in the survey. A total of 3252 students participated out of 4407 total students enrolled across both the Public and Catholic systems, i.e. a total of 72% of enrolled students participated in the survey. The survey data from the EMPATHY project in Red Deer was collected from 3244 participants at baseline (February and March 2014) from three middle schools (serving grades 6–8) and two high schools (serving grades 9–12) in the Red Deer Public School System. For full details of the EMPATHY project, see Silverstone et al. [24, 25]. The primary causes of non-participation were student absences from school during the surveys and logistics constraints. Though Red Deer and Fort McMurray schools devoted substantial resources and staff time to survey data collection, the surveys were done during regular class time, and it was logistically impossible to survey some students due to scheduling conflicts, exams, and so on. Fewer than 50 students in each city chose to decline participation in the survey.

## Data exclusion

Of the 3252 students who participated in the Fort McMurray survey, we excluded 182 because of one or more of the exclusion criteria below. We excluded 448 of the 3244 participants from the Red Deer EMPATHY project, mostly as the majority of the excluded Red Deer participants were in grade 6 and the Fort McMurray survey did not include grade 6 students. Exclusion criteria were as follows:

1. In grade 6
2. Age < = 10 years
3. Age > = 20 years
4. Inconsistent answers among the positive and negative questions from the Rosenberg questionnaire (details in Additional file 1: Appendix 5).
5. Inconsistent answers among the positive questions from the Rosenberg questionnaire and the positive questions from the Kidscreen questionnaire (details in Additional file 1: Appendix 5).
6. Inconsistent answers among the non-reversed and reversed questions from the HADS questionnaire (answer order for two HADS questions is reversed to test for consistency, details in Additional file 1: Appendix 5).

Criteria 4 through 6 above were designed to exclude participants who gave inconsistent answers, possibly because they were not paying attention to the survey or did not understand the questions. After exclusions, there were 3070 participants remaining in the Fort McMurray dataset and 2796 participants remaining in the Red Deer EMPATHY dataset. It is these two datasets that were used for statistical analysis.

## Demographics

Demographics for the 3070 Fort McMurray participants used for statistical analysis were as follows: gender identification was 48% female, 48% male, 2% other, and 2% preferred not to say, and age ranged from 11 to 19, with a mean age of 14.3 and standard deviation 1.8. Demographics for the 2796 Red Deer EMPATHY project participants used for statistical analysis were as follows: gender identification was 48% female and 52% male, and age ranged from 11 to 19, with mean 14.8 and standard deviation 1.7. Means ages for the Fort McMurray and Red Deer students were significantly different (*p* = 0.00001, permutation test, 10^5^ iterations). We ran additional analyses to exclude the possibility that this difference in mean age might have affected the comparisons of interest, and it did not (see end of Statistical Analyses section in the Methods).

## Mental health Indicator comparisons

Comparison of the participant data from Fort McMurray (post-disaster) and Red Deer (no disaster) revealed significant differences in 8 of the 12 measures tested (see Table 2). PHQ-A depression scores were significantly higher in Fort McMurray, as were rates of probable depression, probable moderately severe depression, suicidal thinking, and tobacco use. Rosenberg self-esteem scores and Kidscreen quality of life scores were significantly lower in Fort McMurray. HADS anxiety scores were significantly higher in Fort McMurray, but rates of probable anxiety were not significantly different between the two cities. CRAFFT alcohol / substance misuse scores were also not significantly different, nor were rates of probable alcohol/substance use disorder. In addition, rates of “any of 3 probable diagnoses” were not significantly different. Each comparison included only data from participants who completed all questions in the relevant questionnaires. Numbers of students ranged from 2970 to 3070 in the Fort McMurray group, in comparison to 2796 (consistent across all questionnaires) in the Red Deer group.

**Table 2:** – Fort McMurray survey post-wildfire data vs. Red Deer EMPATHY survey control data

Measure	Fort McMurray Score	Red Deer Score	*P*-value	Effect size
PHQ-A score	8.0 +/− 6.5	5.6 +/− 5.6	0.00001*	0.40
HADS score	7.7 +/− 4.7	7.4 +/− 5.0	0.021*	0.06
CRAFFT score	0.55 +/− 1.25	0.54 +/− 1.16	0.75	0.01
Rosenberg score	18.2 +/− 6.6	20.5 +/− 5.9	0.00001*	−0.36
Kidscreen score	27.0 +/− 8.2	29.4 +/− 7.3	0.00001*	−0.32
Measure	Rate	Rate	*P*-value	
Probable depression	31%	17%	0.00001	
Probable moderately severe depression	17%	9%	0.00001*	
Suicidal thinking	16%	4%	0.00001*	
Probable anxiety	27%	27%	0.98	
Probable alcohol / substance use disorder	15%	16%	0.29	
Tobacco use	13%	10%	0.00090*	
Any of 3 probable diagnoses	37%	36%	0.50	

* *p*-value survives FDR multiple comparison correction (threshold 0.021) Scores are means +/− standard deviation (not standard error)

PTSD symptom data from the CPSS questionnaire was available only for the Fort McMurray group. For that group, CPSS scores had a mean of 12.8 with standard deviation 11.5. Among Fort McMurray students, 37% met conditions for probable diagnosis of PTSD based on thresholding CPSS scores with a cutoff of 15 [38]. CPSS data were not collected for the Red Deer group.

## Discussion

This study compared survey data collected from grade 7–12 students in the disaster impacted community of Fort McMurray and the non-disaster impacted community of Red Deer, in order to examine the effects of disasters on the mental health of adolescents. Consistent with our hypotheses, we observed higher mean PHQ-A scores for depression symptoms, rates of probable depression, suicidal thinking, and tobacco use among grade 7–12 students in the disaster impacted community of Fort McMurray in comparison to the non-disaster impacted community of Red Deer. Rosenberg self-esteem scores and Kidscreen quality of life scores were also lower in the Fort McMurray student population compared to the Red Deer student population. These findings suggest that the wildfire had significant adverse effects on the mental health of adolescents in Fort McMurray due to the traumatic nature of disasters. These results are consistent with the literature which reports a negative impact of wildfire disasters on mental health [7, 9, 10, 12–15, 39–42]. These findings emphasize the need for mental health policies, programs, and supports specifically targeted to adolescents following disaster in order to reduce their vulnerabilities and build positive mental health.

Rates of probable anxiety and probable alcohol/substance use disorder were very similar between Fort McMurray and Red Deer students, counter to our original hypotheses. This was unexpected as population mental health trends for depression tend to match those for anxiety and alcohol/substance use disorder [16]. Fort McMurray Public and Catholic Schools put in place substantial mental health support programming for students after the 2016 wildfire. (See Additional file 1: Appendix for list of mental health support programs.) These results are consistent with the effectiveness of the mental health supports in reducing rates of probable anxiety and alcohol/substance use disorder in the aftermath of the wildfire, though we do not have the pre-fire data necessary to fully support this argument quantitatively. Another possibility (not mutually exclusive) is that these findings may suggest that experiencing a disaster has less impact on adolescents’ rates of probable anxiety and probable alcohol/substance use in the short term (given that the Fort McMurray data was collected just 18 months after the wildfire). The literature indicates that psychological impacts of disaster occur both in the short-term (immediately after evacuation and in the 2 to 5 years rebuilding phase), but also persist in the long-term (up to 10 years during the recovery phase) [43]. These findings highlight the need for a wide range of mental health supports for adolescents following disasters which address both current and potential long-term psychiatric symptoms, in order to support positive mental health among adolescents.

We believe that the Red Deer data provides a compelling community control group given the similarities between communities. Nonetheless, this data was not specifically collected with this intent. A possible issue could be that, while Red Deer experienced no natural disasters prior to or during the survey period, it is conceivable that the 3 year difference in the time of the surveys may have had a minor impact due to some other effect. However, the 2016 wildfire had such a large impact on Fort McMurray and the findings presented here are so striking that we believe any effect from the 3 year difference in survey times would be much smaller than the effects of the 2016 wildfire. In addition, it would have been useful to compare data for symptoms related to disaster trauma exposure between the Fort McMurray and Red Deer samples. This was not possible as we did not have such data from the Red Deer sample.

In conclusion, the current results support existing findings which indicate that adolescents are vulnerable to, and adversely impacted by disasters. The present data extends this by comparing the mental health of a large sample (*n* = 3070) of grade 7–12 students in the disaster impacted community of Fort McMurray to a similarly large sample (*n* = 2796) of adolescents in the non-disaster impacted community of Red Deer, identifying those in Fort McMurray as more vulnerable and at greater risk of low mental health. (To mitigate this, Fort McMurray Public and Catholic Schools have made significant and ongoing efforts to put in place mental health support programs for their students in the aftermath of the wildfire.) The results of this study highlight the importance of longer-term support for students impacted by disasters, particularly focused on depressive symptoms.

## Additional file

Additional file 1: Appendix – Survey Description Script includes a copy of the script read to each class before survey data collection in Fort McMurray. Appendix – Details of Exclusion Criteria contains additional details of the exclusion criteria used for excluding some participants’ data from the analysis, as described in the “Data Exclusion” section in the main text. Appendix – Mental Health Support Programs includes a list of mental health support programs put in place by the Fort McMurray Public and Catholic Schools after the 2016 wildfire. (DOCX 15 kb)

## Abbreviations

CPSS: Child PTSD Symptom Scale
CRAFFT: CRAFFT Questionnaire (proper name of the questionnaire is CRAFFT)
CYRM-12: Child and Youth Resilience Measure
EMPATHY: Empowering a Multimodal Pathway Towards Healthy Youth project
FDR: False discovery rate
HADS: Hospital Anxiety and Depression Scale
Kidscreen-10: Kidscreen Questionnaire
PHQ-A: The Patient Health Questionnaire, Adolescent version
PTSD: post-traumatic stress disorder
Rosenberg: Rosenberg Self-Esteem Scale
