# Correction to: After the Fort McMurray wildfire there are significant increases in mental health symptoms in grade 7–12 students compared to controls

**DOI:** 10.1186/s12888-019-2074-y

**Published:** 2019-03-26

**Authors:** Matthew R. G. Brown, Vincent Agyapong, Andrew J. Greenshaw, Ivor Cribben, Pamela Brett-MacLean, Julie Drolet, Caroline McDonald-Harker, Joy Omeje, Monica Mankowsi, Shannon Noble, Deborah Kitching, Peter H. Silverstone

**Affiliations:** 1 Department of Computing Science, University of Alberta, Edmonton, Canada; 2 Department of Psychiatry, University of Alberta, Edmonton, AB T6G 2B7 Canada; 3 Alberta School of Business, University of Alberta, Edmonton, Canada; 4 Faculty of Social Work, University of Calgary, Calgary, Canada; 5 Department of Sociology and Anthropology, Mount Royal University, Calgary, Canada; 6 Fort McMurray Catholic School District, Fort McMurray, Canada; 7 Fort McMurray Public School District, Fort McMurray, Canada

**Correction to: BMC Psychiatry (2019) 19:18**

**https://doi.org/10.1186/s12888-018-2007-1**

After publication of the original article [1], the authors reported an error in Table 2.

The Probable depression value on the 8th row of the table should have an asterisk as follows:

Probable depression 31% 17% 0.00001 *

This is to indicate that this *p*-value is statistically significant after multiple comparison correction.
